# The Role of Music in Treating Psychiatric Symptoms

**DOI:** 10.1192/j.eurpsy.2022.81

**Published:** 2022-09-01

**Authors:** U. Volpe

**Affiliations:** Università Politecnica delle Marche, Unit Of Clinical Psychiatry, Department Of Clinical Neurosciences/dimsc, Ancona, Italy

**Keywords:** music therapy, psychosis, severe mental illnesses

## Abstract

Music therapy can be defined as the controlled use of music or musical elements by a qualified therapist with a client or a group of clients. The active or passive delivery of musical therapy may facilitate the development of individual potential and/or restore psychological functions of the individual, allowing to obtain better interpersonal, physical, and psychological functioning. Indeed, existing literature suggested that music therapy holds a significant therapeutic potential in a number of psychiatric disorders, including psychosomatic, anxiety and affective syndromes. More recently, evidence concerning the potential of music as a mean to increase group cohesion, acceptance, interpersonal relationships in psychiatric settings has highlighted the potential to improve the patient’s global functioning, social functioning, mental state, and positive/negative symptoms of psychoses. Traditionally, music therapy is delivered in controlled outpatient setting and few evidence point to a possible role in the treatment of acute psychoses, during their hospital stay. Recently, newer evidence has recently piled up and showed that music therapy can induce clinical (in particular, on affective symptoms), functional and quality of life improvement in patients with acute psychoses, even over a short period of time such as during emergency hospitalization. The reported effects might be related to complex neural modulation phenomena involving different interhemispheric, cortical and subcortical brain pathways. Practical clinical experiences, setting or implementation issues and quality standards in music therapy will also be discussed.

**Disclosure:**

No significant relationships.

